# Androgen deprivation and cognition in prostate cancer

**DOI:** 10.1038/sj.bjc.6601235

**Published:** 2003-09-09

**Authors:** E Salminen, R Portin, J Korpela, H Backman, L-M Parvinen, H Helenius, M Nurmi

**Affiliations:** 1 Department of Oncology, Turku University Hospital, Kiinamyllynkatu 4-8, Fin-20520 Turku, Finland; 2 Department of Neurology, University of Turku and University Hospital, Turku, Finland; 3 Department of Surgery, Turku University Hospital, Kiinamyllynkatu 4-8, Fin-20520 Turku, Finland; 4 Department of Biostatistics, University of Turku, Turku 20520, Finland

**Keywords:** prostate cancer, androgen deprivation, cognition, quality of life

## Abstract

Androgen deprivation (AD) is commonly used in neoadjuvant and adjuvant setting with prostate cancer (PC) radiotherapy. This prospective study assessed whether cognitive functioning is impaired during 12 months of AD therapy. Longitudinal testing of 25 patients treated with AD and curative radiotherapy was undertaken at baseline, and at 6 and 12 months. CogniSpeed^©^ software was used for measuring attentional performances. Other cognitive performances were evaluated using verbal, visuomotor and memory tests. The Beck depression inventory was employed to evaluate depressive mood and EORTC QLQ-C30 for quality of life (QoL). During longitudinal testing of the AD group, no impairment in cognitive performances was found. Instead, improvement was observed in object recall (immediate, *P=*0.035; delayed, *P*<0.001), and in semantic memory (*P=*0.037). In QoL, impairment in physical function was observed. Androgen deprivation of 12 months appears to be associated with preserved cognitive functioning, although physical impairment occurs. These results have implications for counseling and psychosocial support of patients in the context of treatment options in PC.

Prostate cancer (PC) has become one of the most common cancers among men in the Western world. Most patients are diagnosed with potentially curable disease. During recent years, it has become common practice to combine androgen deprivation (AD) with radical radiotherapy in a neo+adjuvant setting, since this approach has been shown to improve survival of patients with poor prognosis (Bolla *et al*, 1997). A striking increase in the incidence of PC with poor prognosis in men aged <60 years has been reported (Post *et al*, 1999). A combined approach has been suggested to reduce the mortality due to this disease (Demers *et al*, 2001). Androgen deprivation has also been proposed as an alternative to watchful waiting for men with clinically localised disease electing less aggressive management (Yang *et al*, 1998), and AD is widely used as a means of preventing progression if local therapies cannot be utilised for reasons of patient's preference or poor general condition (Gee, 1995).

Although AD is the treatment of choice in metastatic PC, there is no international consensus on indications or treatment duration in the use of neo+adjuvant treatment in PC. The indications for and duration of AD used in connection with radical treatment call for evidence-based recommendations. Any treatment of these patients causing adverse effects should therefore be carefully monitored to ensure that treatment-related effects do not exceed the disease-related (Fransson *et al*, 2001). A common practice outside clinical trials is to apply AD for 3–6 months, but treatments as long as several years are also practised. The use of AD appears to be most common in early-stage disease (T1–T2), as reported by Potosky *et al* (2001), these being patients who are usually long-term survivors in PC. At least 22 000 PC men each year in the US have been estimated to receive hormonal therapy (Potosky *et al* 2001).

Androgen deprivation therapy for PC is known to affect the quality of life (QoL) adversely, leading to increased fatigue, erectile difficulties and decline in sexual function (Potosky *et al*, 2002). Although cognitive complaints are not uncommon during AD treatment (Green *et al*, 2002a,2002b), they have not hitherto been systematically addressed in this patient group. There have been suggestions that lowered testosterone level is associated with impairment of cognitive functioning (O'Connor *et al*, 2001) and that testosterone replacement therapy may improve certain memory performances (Cherrier *et al*, 2001), but these studies have addressed on healthy old men. Whether AD affects cognitive functioning in newly diagnosed PC patients in any significant way has not, to our knowledge, been previously reported. We hypothesised that AD may have an impact on cognitive performances in PC patients, especially on attention and memory, because these functions are known to be sensitive to disease-related and other kinds of stress effects (Barrett-Connor *et al*, 1999). A systematic study on cognitive functioning during 1 year of AD treatment of PC patients was conducted to study quantitatively the changes associated with this treatment.

## PATIENTS AND METHODS

### Patients

Extensive cognitive testing was undertaken on three occasions in 25 men receiving neo+adjuvant AD therapy for 12 months in connection with radical radiotherapy for PC. The patients had to fulfil at least two of the following criteria to be eligible for hormonal treatment: tumour grade >2, Gleason >5 and Prostate-specific antigen (PSA) >20. Only patients with newly diagnosed PC requiring therapy were accepted. Eight patients (32%) had performance status 0 and 17 patients (68%) status 1 as measured by WHO criteria (Miller, 1981). World Health Organisation (WHO) performance status 0 indicates full functioning with no symptoms, and WHO 1 indicates normal performance status with slight or occasional symptoms. Most of the patients had a T3 tumour (84%); there were two with T2 and two with T4 tumours, and all represented grade 2–3 tumours. Their mean PSA at diagnosis was 31 ng l^−1^ (s.d. 25.4). None of the patients developed progressive disease during the 12-month study period.

The 52 healthy controls for baseline evaluation were voluntary male participants who had no psychiatric or neurological disorders or history of drug or alcohol abuse. Control group of normal healthy men at baseline was used to comparison of basic cognitive performances of the PC patient group. The patients and control persons came from southwestern Finland and were matched for distribution of age and education. The mean age of the patients was 64.4 years (s.d. 6.5, range 49–75), and their mean education 8.9 years (s.d. 2.9, range 6–15). The mean age of the controls was 65.3 years (s.d. 6.6, range 46–77), education a mean of 8.5 years (s.d. 2.1, range 6–15). Mini-mental state examination (MMSE) was performed: for the patients, the mean score was 27.1 (s.d. 2.0, range 21–29), and for the controls 28.0 (s.d. 1.4, range 25–30). There was no statistically significant difference between the groups; two patients who had a lower score than controls were accepted.

To be eligible for neuropsychological testing, patients had to meet the following inclusion criteria: (1) no evidence of progressive or metastatic disease, (2) no history of neurological/psychiatric signs or symptoms that might lead to deviant neuropsychological test results, (3) no abuse of alcohol or drugs and (4) the mother tongue being Finnish. Written informed consent was obtained from all patients. The study was approved by the joint ethical committee of Turku University Hospital and the University of Turku.

Androgen deprivation therapy was started with flutamide given for 4 weeks, and LHRH analogue (s.c. q 3 months four times) was added after 2 weeks. Thus, AD therapy lasted 12 months. Radiotherapy was given by the conformal technique using the 15 MV photons/Varian Clinac 2100C/D linear accelerator (Varian Inc. Palo Alto, CA, USA) to a mean tumour dose of 69 Gy (s.d. 3.15, range 61–77 Gy). No fixation was used. Portal images were taken with a Varian Portal Vision Mark 2 electronic portal imaging device, integrated with Varis Vision 5.0 software, on average once a week during about 7-week irradiation.

### Neuropsychological testing

Neuropsychological testing was conducted using a cognitive test battery at baseline, and at 6 and 12 months. Individuals in the control group were examined only at baseline. The patients acted as their own controls in the follow-up. The patients also attended for clinical control check-up every 6 months after the onset of therapy and were interviewed with regard to physical and mental symptoms as experienced in daily life; PSA, Hb, serum creatinine and liver enzymes were taken and QoL forms were filled on the same occasions.

Our study was exploratory because there were no earlier reports of antiandrogen effects on cognition in newly diagnosed PC patients. The methods with corresponding references are explained in full detail in the Appendix. We selected an extensive and systematic test battery to evaluate basic verbal, visuomotor and memory domains, and overall level of performances, as well as cognitive speed and accuracy. Attentional domains and memory can be expected to be particularly sensitive to both disease- and treatment-related effects.

Cognitive processing was studied using CogniSpeed^©^ software, planned to measure both automatic (well-learned) and controlled, attention demanding processing. Automatic tasks consist of recognition of familiar items (numbers and letters), whereas tasks of controlled processing demand working memory or sustaining attention. These functions may be most vulnerable to harmful effects, such as depletion of hormone concentrations. Attention and memory performances may reflect cognitive processing efficiency also in everyday situations. One purpose of the study was to find out whether there are cognitive domains sensitive to impair in response to a year-lasting lack of androgens.

In order to utilise all information provided by test variables, we used original data as continuous, not categorised variables. To ensure that the subject had capacity to improvement, we evaluated a subject's overall impairment on verbal, visuomotor and memory performances, using standard deviations of an independent norm group. Most patients performed as controls on this deterioration scale (range 0–18 points), which provides more information than MMSE: for the controls the range was 0–3 (mean 0.80) and for the patients 0–4 (mean 0.84) (Table 1

**Table 1:** Verbal, visuomotor and memory performances in patients (*n*=25) and controls (*n*=52): comparison at baseline, and testing of patients from baseline through 12 months (means (s.e.'s))

			**Patients**				
	**Controls**	***P*-value Controls/patients**	**Baseline**	**6 months**	**12 months**	***P*-value Total period**	**Pairwise comparisons *P*<0.05^a^**
Similarities	19.9(0.39)	0.511	20.2 (0.64)	20.2 (0.65)	21.6 (0.66)	0.037	6<12
Digit span	10.9 (0.30)	0.008	9.5 (0.32)	10.1 (0.33)	10.5 (0.33)	0.062	
Digit symbol	37.3 (1.25)	0.011	30.9 (2.03)	31.5 (2.07)	34.2 (2.05)	0.166	
Block design	32.4 (1.12)	0.497	30.0 (1.73)	31.3 (1.74)	31.7 (1.75)	0.260	
Object naming (s)	33.7 (1.23)	0.656	36.3 (2.23)	37.1 (2.29)	33.5 (2.27)	0.316	
Object recall (immediate)	12.1 (0.32)	0.412	12.4 (0.45)	13.6 (0.47)	13.8 (0.46)	0.035	B<6
Object recall (delayed)	10.1 (0.31)	0.927	10.4 (0.56)	11.9 (0.57)	12.3 (0.56)	0.001	B<6;B<12
Deterioration score	0.80 (0.28)	0.922	0.84 (0.25)	0.94 (0.26)	0.43 (0.26)	0.245	
Verbal fluency^b^	27.3 (2.36)	0.049	21.3 (1.08)	22.0 (1.10)	21.9 (1.08)	0.791	
Picture naming^b^	13.3 (0.58)	0.874	13.4 (0.31)	13.4 (0.32)	13.7 (0.31)	0.281	
Word list recall^b^	19.3 (1.26)	0.242	21.0 (0.78)	21.1 (0.80)	20.8 (0.79)	0.875	
Benton, visual recognition (correct)			5.2 (0.25)	5.6 (0.26)	5.4 (0.26)	0.158	
Visual span (Wechsler memory scale)			15.8 (0.61)	16.0 (0.62)	15.4 (0.62)	0.534	
Mini-mental state	28.0 (0.20)	0.147	27.1 (0.40)	27.5 (0.40)	27.6 (0.40)	0.611	
Beck depression score	2.7 (0.33)	0.780	2.8 (0.53)	2.5 (0.55)	2.2 (0.54)	0.753	

B=baseline; 6=6 month-testing; s.e.=standard error; s=second; 12=12 month-testing;

a Tukey's multiple comparison corrected.

b Number of controls=12.

). With regard to clinical practice, it is always possible to compare an individual score in any test, for example, in reaction time tests, with the means and standard deviations of a healthy control group.

### Quality of life

At the time of cognitive testing, the patients were also asked to fill in EORTC QLQ-C30 forms (Fayers *et al*, 1997) completed with sexual function items. These items in this study included questions about level of sexual interest, ability to achieve and maintain erection, gynaecomastia, spousal relationship and mood. Other items asked respondents about overall physical or mental discomfort, worry, role functioning, limitations in daily activities and bother due to PC or treatments.

### Statistical analyses

The data were summarised showing mean values and standard errors (s.e.). the patients who had measurements at baseline and at least at either of the two follow-up time points were included in the analyses. Statistical comparisons of patients and controls at baseline were made using two-sample *t*-test. Within the patient groups, the comparisons of different time points were carried out with analysis of variance for repeated measurements. The pairwise *post hoc* comparisons of time points were conducted by Tukey's method. The analyses were performed using MIXED procedure in the SAS System for Windows, release 6.12/1996. The MIXED procedure offers a sophisticated tool for analysis of follow-up data with possible missing data during follow-up (Littell *et al*, 1996). *P*-values less than 0.05 were considered to be statistically significant.

## RESULTS

At baseline, the AD group performed at a lower level than the control group, matched for distribution of age and education, in immediate memory of digit span (*P=*0.008), in visuomotor digit symbol (*P=*0.011) and in word fluency (*P=*0.049). Comparisons between controls and patients at baseline are shown in Tables 1 and 2

**Table 2:** Cognitive speed and attention in patients ((*n*=25) and controls (*n*=52): comparison at baseline, and testing of patients from baseline through 12 months (means (s.e.'s))

			**Patients**			
	**Controls**	***P*-value Controls/patients**	**Baseline**	**6 months**	**12 months**	***P*-value Total period**
Simple RT (ms)	282 (4.8)	0.427	291 (10.9)	311 (11.2)	324 (11.3)	0.061
2-CRT (ms)	506 (16.3)	0.545	534 (19.9)	547 (20.1)	532 (20.2)	0.391
2-CRT, errors	1.1 (0.22)	0.106	0.52 (0.17)	0.79 (0.18)	0.56 (0.18)	0.599
10-CRT (ms)	1002 (24.8)	0.056	1113 (48.5)	1134 (49.0)	1130 (49.6)	0.786
10-CRT, errors	0.4 (0.10)	0.534	0.60 (0.20)	0.62 (0.21)	0.81 (0.22)	0.717
Subtraction (ms)	1599 (64.4)	0.064	1968 (154.5)	2017 (155.8)	1871 (157.9)	0.334
Subtraction, errors	1.7 (0.25)	0.120	3.2 (0.80)	3.1 (0.81)	4.1 (0.83)	0.355
Subtraction time	597 (53.4)	0.116	855 (136.5)	917 (138.1)	740 (140.4)	0.220
Vigilance^a^ (ms)	463 (5.6)	<0.001	509 (9.4)	501 (9.6)	504 (10.1)	0.579
Vigilance^a^, errors	2.4 (0.48)	0.115	4.1 (0.76)	2.4 (0.79)	3.7 (0.85)	0.159

*Recognition*						
Numbers^a^ (ms)	62.7 (2.91)	0.200	70.8 (3.14)	69.9 (3.43)	67.3 (3.35)	0.635
Letters^a^ (ms)	67.6 (3.06)	0.393	76.8 (4.17)	73.8 (4.40)	69.7 (4.37)	0.234

SE=standard error; ms=millisecond; RT=reaction time; 2-CRT=two-choice choice reaction time; 10-CRT=10-choice reaction time.

a Number of controls=33.

.

**Figure 1:**
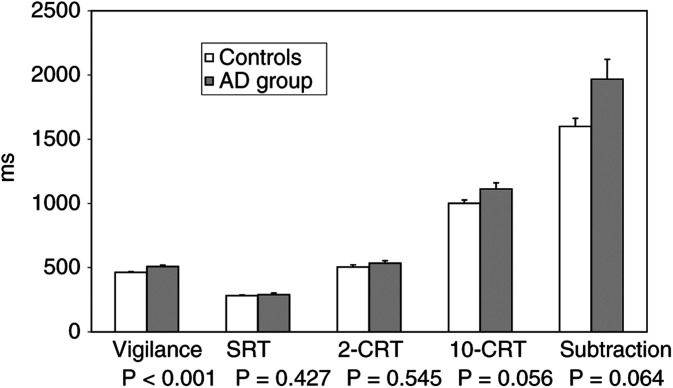
Cognitive performance speed at baseline in reaction time tasks in patients starting AD therapy (AD group) and controls (means (s.e.'s)).

Other performances in memory tests (semantic memory, visual memory, word recall), in visuomotor block design or naming tests did not differ between the groups. The depression scores were low and did not differ. Further, the AD group performed more slowly than the controls in the sustained attention vigilance task (*P*<0.001), but the groups did not differ in the other reaction time tasks or in the recognition task (Table 2). A comparison of the AD group and controls in reaction time tests in different attentional domains is presented in Figure 1

.

**Figure 2:**
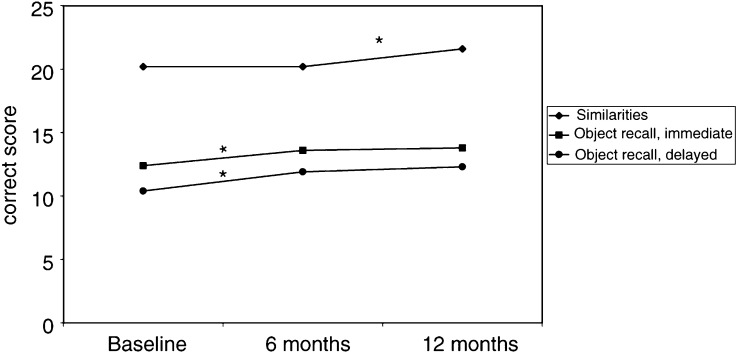
Cognitive performance of patients during AD therapy from baseline through 6 and 12 months in three memory tests; statistically significant improvement, marked as *.

During the period of 12 months on AD, some changes emerged in memory performances compared to the baseline values (Table 1). The patients improved their performances in episodic memory (immediate recall of objects, *P*=0.035, significant change for the first 6 months and delayed recall *P*<0.001, significant change for the whole test period) and in semantic memory (similarities, *P=*0.037, significant change for the latter half of the test period (Figure 2

). Other performances, including reaction speed and accuracy in reaction time tests, remained unchanged through the follow-up testings (Table 2). Beck depression scores remained similar throughout the follow-up testings.

On the self-report QoL forms (Table 3

**Table 3:** Physical scale values during 12 months of androgen deprivation expressed as means and standard errors (s.e.)

	**Baseline**	**6 months**	**12 months**	
**Function scales**	**Mean (s.e.)**	**Mean (s.e.)**	**Mean (s.e.)**	***P*-value**
Physical	87.1 (4.29)	79.3 (4.05)	77.2 (4.52)	0.0004
Cognitive	89.2 (2.21)	86.5 (3.21)	86.8 (2.66)	0.3451
Emotional	82.4 (3.35)	82.7 (3.58)	79.9 (3.76)	0.5919
Social	94.1 (1.98)	87.1 (2.99)	86.8 (4.33)	0.2098
Role functioning	95.7 (2.40)	98.2 (1.79)	95.0 (2.79)	0.3255
Feeling sick	1.07 (0.05)	2.42 (0.18)	2.55 (0.95)	<0.0001

EORTC=European Organization for Research and Treatment of Cancer. Higher score means better functioning, scale 0–100.

) a significant worsening of physical functioning was seen when the results of 12 months were compared with that of baseline.

During the treatment, the patients expressed significantly more suffering from fatigue, dyspnoea and diarrhoea compared to baseline. No statistically significant changes were reported in the case of emotional, social or cognitive functioning. Table 4

**Table 4:** Comparison of selected quality of life items during 12 months of androgen deprivation expressed as means and standard errors (s.e)

**Symptom**	**Baseline**	**6 months**	**12 months**	
**scales/items**	**Mean (s.e.)**	**Mean (s.e.)**	**Mean (s.e.)**	***P*-value**
Fatigue	14.3 (3.08)	19.7 (4.10)	20.5 (4.30)	0.044
Dyspnoea	8.57 (3.70)	13.6 (3.67)	17.2 (4.57)	0.030
Insomnia	21.6 (4.42)	34.6 (4.87)	34.5 (5.36)	0.1071
Diarrhoea	4.76 (2.00)	17.3 (5.15)	17.2 (4.26)	0.001
Erectile dysfunction	221 (1.07)	3.08 (1.06)	3.32 (0.86)	0.0002
Gynaecomastia	1.23 (0.08)	1.46 (0.15)	1.46 (0.12)	0.0005
Libido	1.83 (0.16)	3.00 (0.20)	3.10 (0.18)	<0.0001
Male identity	1.96 (0.96)	3.28 (0.74)	3.00 (1.07)	0.0011
Depressive mood	1.77 (0.16)	1.54 (0.13)	1.77 (0.16)	0.8567
Spouse relationship	1.32 (0.10)	1.64 (0.18)	1.48 (0.16)	0.2332
Urinary frequency	2.25 (0.89)	2.15 (0.73)	2.00 (0.82)	0.0425^a^

a Other items worsened, this item improved significantly.

shows in detail the development of changes in physical and emotional items. Although the patients reported their sexual interest, male identity and erectile function to decrease significantly during AD, they did not feel more depressed nor did they indicate that their relationship with their spouse suffered from their present condition more than at baseline.

## DISCUSSION

Since AD treatment is commonly used in PC, the objective of our study was to investigate the effects of AD at 6 and 12 months on cognitive function in PC patients with a view to helping the clinician in decision-making and education of patients on treatment effects. The current study showed some deficits in verbal and visuomotor performances as well as sustained attention of the patient group in comparison to controls. During AD treatment, in spite of impaired physical function in these patients, they showed significant improvement in some memory performances and no cognitive impairments when AD treatment was given for up to 12 months. During 12-month testing of the AD group, significant improvement was observed compared to baseline values in episodic memory measures of object recall and in semantic memory, whereas no impairment was found in any cognitive performances evaluated, including cognitive speed in reaction time tasks.

To minimise individual variability and learning effect in reaction time tasks, there was a practice round preceding the CogniSpeed^©^ subtests. The repeated measures of CogniSpeed^©^ tests have shown a reliable and steady course over trials in healthy persons and in the study with brain tumour patients (Lilja *et al*, 2001). Furthermore, the six tests of cognitive deterioration, including verbal, visuomotor and memory tests, are sensitive to cognitive impairment, even if the same versions are repeated (Kujala *et al*, 1997). Learning effects normally occur in free recall tasks in repeated measurements. We do not propose that there could not be improvement in performances of controls, if tested, because learning effects normally occur in free recall tasks in repeated measurements. Learning effect can be considered a positive sign of one's capacity to utilise experience rather than a negative source of error.

Androgen deprivation therapy for PC is known to affect the QoL adversely, leading to increased fatigue, erectile difficulties and declines in sexual functioning (Lubeck *et al*, 2001). The duration of AD in clinical practice varies from 3 months to 3 years and even longer. The present study set-up was motivated by the common practice of using AD in the treatment of early-stage PC outside clinical trials. We chose to test 12 months of treatment in view of superior effects shown compared to shorter (Gleave *et al*, 2001), and avoided longer treatment in order to delay the development of hormone-resistant disease (Newling, 2000).

We focused on effects of hormonal treatment on attention and information processing as well as memory function as these domains of cognition are considered vulnerable to changes in sexual hormone level (Barrett-Connor *et al*, 1999). In the literature, there are some indications that androgen treatment may modulate aspects of cognitive performance (Wolf *et al*, 2000). It has also been suggested that testosterone replacement therapy may have a positive effect on cognition, in particular on improved spatial and verbal memory in healthy older men (O'Connor *et al*, 2001). This is partly in agreement with the present results, which showed selective improvement in episodic and semantic memory measures, whereas attention and information processing speed remained unchanged through the treatment period. On the other hand, decreased testosterone levels may be associated with impaired cognition in aging men (Cherrier *et al*, 2001). In the present study, the patients performed some tasks at lower level than the control persons of similar age and education, when the groups were compared at baseline before the start of antiandrogen therapy. Thus, this impairment may refer to disease-related rather than to more specific effects. Longitudinal cognitive stability and improvements may suggest some positive hormonal influence on brain functioning.

We recognise some limitations in the interpretation of our results. We used normal controls at baseline as a reference group for the PC patients. However, the groups were matched for basic characteristics: they were of similar age and education, with no major diseases. In the longitudinal study, the effect of AD treatment on cognition was evaluated using the patient's own baseline values. This strengthens the interpretation of results, while elderly PC patients are a heterogenous patient group with often several comorbidities that could affect the results of cognitive tests. Our results with similar pattern of cognitive testing with patients treated with curative radiotherapy at the same dose level as the study patients without AD resulted in no statistically significant changes in cognition (Salminen *et al*, 2002).

In concord with the observations of Van Dam *et al* (1998) in chemotherapy-treated breast cancer patients, we observed that patients complained, for example, of depressive mood and similar discomforts, although these were not confirmed by the Beck depression results. It has been proposed that objective test results and subjective reports of patients regarding their cognitive functioning and mood are not always related; there has been no relationship between the self-reported difficulties and the performance of patients in objective tests (Cull *et al*, 1966; Ly *et al*, 2001). Furthermore, PC patients with nonlocal cancer randomised either to AD or follow-up did not differ in subjective cognitive function (Green *et al*, 2002a,2002b). Subjective reports have been found to be related to anxiety and depression, being indicative more of emotional distress than cognitive deficits (Van Dam *et al*, 1998; Ly *et al*, 2001).

In comparison to a report by Fransson *et al* (2001), who observed decreased QoL in the social functioning of PC patients treated with radiotherapy only, our patients did not evince this, although the forms they filled in were similar. The difference is in evaluation time, which was over 3 years in Fransson's series. The deterioration in physical function reported by our patients was in line with the previous results (Lubeck *et al*, 2001).

## CONCLUSION

Healthy PC patients treated with AD in connection with curative radiotherapy maintain their cognitive functioning. Cognitive deficits found at baseline were associated with disease severity rather than with treatment-related factors. The documentation of cognitive function has substantial implications for informed patient's support when hesitating between treatment options. A longer follow-up study with cognitive assessment is needed if AD is extended for several years, and further studies are needed to clarify the effects of AD in patients with severe comorbidity.

## Appendix

Cognitive tests were used to measure verbal, visuomotor and memory performances, as well as cognitive processing in different attentional domains. The investigations were performed in 3-h sessions, with short breaks if needed, by two experienced psychologists.

Two verbal and two visuomotor subtests (Wechsler, 1955) were administered: similarities, digit span, digit symbol and block design. The verbal fluency test comprised generating orally names of animals during 1 min. In the picture-naming test, the subject had to name 15 line drawings presenting concrete objects.

Episodic memory was investigated using four tests: (1) naming time, immediate and delayed (after 1 h) recall of 20 common objects (Lilja *et al*, 2001); (2) the word list recall consisted of 10 words, which the subject was instructed to read when shown to him/her one at a time, and there were three trials for immediate recall (Lezak, 1995); (3) the visual recognition test consisted of seven designs (form C), shown one at a time for 10 s (Benton, 1963). After the study phase, the set was removed and the subject had to point to the right design among four alternatives; (4) visual memory span (Wechsler, 1987) comprised tests of tapping squares on a card in a given order. The total score was the sum of correctly repeated series forward and backward.

The overall cognitive deterioration score consisted of deterioration points in six tasks addressing memory, visuomotor and verbal domains (similarities, digit span, digit symbol, block design, naming time and immediate recall of 20 objects). The results on these tests were rescored based on our earlier results obtained by healthy subjects. The subjects received one deterioration point if their performance in any of the tests was below 1.5 s.d. compared to the norms, two points if below 2 s.d., and three points if below 3 s.d. The maximum deterioration score was thus 18 points (Kujala *et al*, 1994).

To rule out severe cognitive impairment, the MMSE was administered (Folstein *et al*, 1975); the cutoff was set as 21 points. The Beck depression inventory was administered to evaluate depressive mood (Beck *et al*, 1974).

The CogniSpeed^©^ software was used to measure the speed and accuracy of automatic and controlled cognitive processing (Kujala *et al*, 1994; Revonsuo *et al*, 1995). Reaction times (RTs) were measured in milliseconds (ms) in correct responses, and the error scores were recorded. In each CogniSpeed^©^ test, a practice session was held before the final test round, and the subject was instructed to respond as quickly as possible. The final test rounds comprised 40 items.

Automatic processing was studied with a task involving recognition thresholds of well-learned targets (four numbers and six letters). A letter ‘X’ shown in the middle of the screen was replaced briefly by the target. The first presentation time of 14 ms was increased stepwise in 14-ms steps, until the target was identified.

Controlled processing addressing different attentional functions, was evaluated in series of RT tasks, which became more difficult step by step. In the simple reaction time (SRT) test, the subject had to press the ‘0’ key every time the target ‘0’ appeared on the screen with a random delay ranging from 1 to 4 s. In the choice RT tests, the stimuli appeared in random order. In the two-choice (2-CRT) test, either ‘1’ or ‘2’ appeared in the middle of the screen and the subject was instructed to press the corresponding key. In the 10-choice (10-CRT) test, the numbers 0–9 appeared in the middle of the screen, and the subject had to press the corresponding number. The subtraction test, which requires concentrating attention and working memory, was identical to the 10-CRT test, but the instruction given to the subject was different: the number appearing on the screen had to be subtracted from nine, and the key corresponding to the remainder pressed. The subtraction time was the difference in the RTs between the subtraction test and the 10-CRT test, with similar perceptual and motor requirements. Thus, subtraction time represented conscious working memory components in tasks involving relatively high attentional demands.

The vigilance test of letter cancellation measured sustaining attention. This was a monotonous task of 15 min, with target events occurring at a relatively slow rate and in random order. There were two target letters (Y, L) appearing with a probability of 20% among all 600 letters; the presentation time was 500 ms and the interval between letters 1000 ms.

## References

[bib1] Barrett-Connor E, Goodman-Gruyen D, Patay B (1999) Endogenous sexual hormones and cognitive function in older men. J Endocrinol Metab 84: 3681–3685 10.1210/jcem.84.10.6086 10523014

[bib2] Beck AT, Rial WY, Rickels K (1974) Short form of depression inventory: cross-validation. Psychol Rep 34: 1184–1186 4424377

[bib3] Benton AL (1993) Revised Visual Retention Test. New York: Psychological Corporation

[bib4] Bolla M, Gonzalez D, Worde P, Dubois J, Minmanoff RO, Storme K, Bernitz J, Kuhen A, Sternberg C, Gil T, Collette L, Plerart M (1997) Improved survival in patients with locally advanced prostate cancer treated with radiotherapy and goserelin. N Engl J Med 337: 295–300 9233866 10.1056/NEJM199707313370502

[bib5] Cherrier M, Anwalt B, Herlst K, Amory J, Craft S, Matsumoto AM, Brvemmer WJ (2001) Testosterone supplementation improves spatial and verbal memory in healthy older men. Neurology 10: 80–88 10.1212/wnl.57.1.80 11445632

[bib6] Cull A, May C, Love S, Mackie M, Smets E, Stewart M (1966) What do patients mean when they complain of concentration and memory problems? Br J Cancer 74: 1674–1679 10.1038/bjc.1996.608 PMC2074867 8932354

[bib7] Demers R, Tiwari A, Wei J, Wein Z, Serenson R, Montie J (2001) Trends in utilization of androgen-deprivation therapy for patients with prostate carcinoma suggest an effect on mortality. Cancer 92: 2309–2317 11745285 10.1002/1097-0142(20011101)92:9<2309::aid-cncr1577>3.0.co;2-8

[bib8] Fayers P, Aaronson N, Bjordal K, Sullivan M (1997) EORTC QLQ-C30 Scoring Manual. Brussels: EORTC Publications

[bib9] Folstein MF, Folstein SE, McHugh PR (1975) ‘Mini Mental State’: a practical method for grading the cognitive state of patients for clinician. J Psychiatr Res 12: 189–198 1202204 10.1016/0022-3956(75)90026-6

[bib10] Fransson P, Dambü J, Tomic R, Modig H, Nyberg G, Widmark A (2001) Quality of life and symptoms in a randomized trial of radiotherapy versus deferred treatment of localized prostate carcinoma. Cancer 92: 3111–3119 11753990 10.1002/1097-0142(20011215)92:12<3111::aid-cncr10160>3.0.co;2-e

[bib11] Gee WF (1995) Practise trends in the diagnosis and management of prostate cancer in USA. J Urol 154: 207–208 7539862

[bib12] Gleave M, Goldenberg S, Chin J, Warner J, Saad F, Klotz L, Jewett M, Kassalian V, Chetner M, Dupont C, Van Reusselaer S, the Canadian Uro-Oncology Group (2001) Randomized comparative study of 3 versus 8-month neoadjuvant hormonal therapy before radical prostatectomy: biochemical and pathological effects. J Urol 166: 500–507 11458055

[bib13] Green H, Palanham K, Headley B, Gardiner R (2002a) Altered cognitive functioning in men treated for prostate cancer with luteinizing hormone-releasing hormone analogues and cyprotenone acetate: a randomized controlled trial. BJU Int 90: 427–432 12175403 10.1046/j.1464-410x.2002.02917.x

[bib14] Green H, Palanham K, Headley B, Gardiner R (2002b) Coping and health-related quality of life in men with prostate cancer randomly assigned to hormonal medicationor close monitoring. Psycho-oncology 11: 401–414 12228873 10.1002/pon.599

[bib15] Kujala P, Portin R, Revonsuo A, Ruukainen J (1994) Automatic and controlled information processing in multiple sclerosis. Brain 117: 1115–1126 7953593 10.1093/brain/117.5.1115

[bib16] Kujala P, Portin R, Ruutiainen J (1997) The progress of cognitive decline in multiple sclerosis – a controlled 3-year follow-up. Brain 120: 289–297 9117376 10.1093/brain/120.2.289

[bib17] Lezak MD (1995) Neuropsychological assessment, 3rd edn. New York: Oxford University Press

[bib18] Lilja A, Portin R, Hamalainen P, Salminen E (2001) Short-term effects of radiotherapy on attention and memory performances in patients with brain tumours. Cancer 91: 2361–2368 11413526

[bib19] Littell R, Milliken GA, Stroup W, Wolfinger RD (1996) SAS® System for Mixed Models. Cary, NC: SAS Institute Inc.

[bib20] Lubeck D, Grossfeld G, Carroll P (2001) The effect of androgen deprivation therapy on health-related quality of life in men with prostate cancer. Urology 58(Suppl 2a): 94–100 11502459 10.1016/s0090-4295(01)01250-x

[bib21] Ly L, Juminez M, Huang T, Celermayer D, Conway A, Handesman D (2001) A double-blind, placebo controlled, randomized clinical trial of transdermal dihydrotestosterone gel on muscular strength, mobility, and quality of life in older men with partial androgen deficiency. J Clin Endocrinol Metab 86: 4078–4088 11549629 10.1210/jcem.86.9.7821

[bib22] Miller AB, Hoogstraten B, Staguet M, Winkler A (1981) Reporting results of cancer treatment. Cancer 47: 201–214 10.1002/1097-0142(19810101)47:1<207::aid-cncr2820470134>3.0.co;2-6 7459811

[bib23] Newling D (2000) Tailoring of hormonal therapy in prostate cancer. Prostate Cancer PD 3: 21–27 10.1038/sj.pcan.4500400 12497157

[bib24] O'Connor D, Archer J, Hair W, Wu F (2001) Activational effects of testosterone on cognitive function in men. Neuropsychology 39: 1385–1394 10.1016/s0028-3932(01)00067-7 11585606

[bib25] Post P, Stockton D, Davies D, Coebergh J (1999) Striking increase in incidence of prostate cancer in men aged <60 years without improvement of prognosis. Br J Cancer 79: 13–17 10408686 10.1038/sj.bjc.6690004 PMC2362175

[bib26] Potosky A, Knopf K, Clegg L, Albertsen P, Stanford J, Hamilton A, Gilliland F, Elev J, Stephenson R, Hoffman R (2001) Quality of life outcomes after primary androgen deprivation therapy: Results from the prostate cancer outcomes study. J Clin Oncol 19: 3750–3757 11533098 10.1200/JCO.2001.19.17.3750

[bib27] Potosky A, Reeve B, Clegg L, Hoffman R, Stephenson R, Albertsen P, Gilliland F, Stanford J (2002) Quality of life following localized prostate cancer treated initially with androgen deprivation therapy or no therapy. J Natl Cancer Inst 94: 430–437 11904315 10.1093/jnci/94.6.430

[bib28] Revonsuo P, Portin R (1995) CogniSpeed^©^ The Computer Based Measurement of Cognitive Processing. University of Turku, Turku, Finland: Aboa Tech Ltd

[bib29] Salminen E, Lempainen L, Nurani M, Baerman H, Parunen LM, Portin R (2002) Investigation on androgen deprivation with radical radiotherapy of prostate cancer. In Progress in Radio-Oncology, vol. 11, Kogelnik H, Lukas P, Sedlmayer F (eds) pp 75–81. Bologna, Italy: Monduzzi Editore

[bib30] Van Dam F, Schlagen S, Muller MG, Boogard W, Wall E, Droogleever-Fortujn M, Rodenhuis S (1998) Impairment of cognitive function in women receiving adjuvant treatment for high risk breast cancer: high-dose versus standard-dose chemotherapy. J Natl Cancer Inst 90: 210–218 9462678 10.1093/jnci/90.3.210

[bib31] Wechsler D (1955) Manual for the Wechsler Adult Intelligence Scale. New York: Psychological Corporation

[bib32] Wechsler D (1987) WMS-R, Wechsler Memory Scale-Revised. Manual. New York; Psychological Corporation, Harcourt Brace Jovanovich, Inc.

[bib33] Wolf O, Hellhammer D, Kudillea B, Schurmeyer T, Kirschbaum C (2000) Testosterone and cognition in elderly men: a single testosterone injection blocks the practice effect in verbal fluency, but has no effect on spatial or verbal memory. Biol Psychiatry 47: 650–654 10745058 10.1016/s0006-3223(99)00145-6

[bib34] Yang FE, Song PY, Wayne J, Vaida F, Vijayakumar S (1998) A new look at an old option in the treatment of early-stage prostate cancer: hormone therapy as an alternative to watchful waiting. Med Hypotheses 51: 243–251 9792203 10.1016/s0306-9877(98)90083-4

